# Association between radiotherapy and prognosis in patients with small cell carcinoma of the bladder undergoing bladder-sparing surgery

**DOI:** 10.3389/fonc.2023.1275796

**Published:** 2023-10-17

**Authors:** Fuchao Liang, Fei Zhou, Xiaoyuan Qian, Linghui Qin, Jiange Wang, Chen Ding, Yasen Kuniduzi, Xuejun Zhang, Lianming Fan

**Affiliations:** ^1^ Department of Urology, Xiangyang Central Hospital, Affiliated Hospital of Hubei University of Arts and Science, Xiangyang, Hubei, China; ^2^ College of Medicine, Wuhan University of Science and Technology, Wuhan, Hubei, China

**Keywords:** small cell carcinoma of the bladder, radiotherapy, bladder-sparing surgery, cancer-specific survival, SEER

## Abstract

**Background:**

Small cell carcinoma of the bladder is rare and has a poor prognosis. This study aimed to investigate whether radiotherapy after bladder-sparing surgery could improve the survival benefits of patients.

**Methods:**

This population-based retrospective cohort study used data from the Surveillance, Epidemiology, and End Results cohort in the United States to investigate small cell carcinoma of the bladder. Univariate and multivariate Cox regression analyses were used to identify significant risk factors influencing the clinical prognosis. A propensity score matching (PSM) algorithm was used to reduce the interference of confounding factors in each study group. The matched groups underwent Kaplan–Meier survival analysis to assess the potential survival benefits.

**Results:**

Univariate regression analysis demonstrated that age (P<0.001), tumour stage (T stage) (P=0.005), node stage (N stage) (P<0.001), chemotherapy (P<0.001), bone metastasis (P<0.001), liver metastasis (P<0.001), lung metastasis (P=0.005), tumour size (P=0.005), and radiotherapy (P<0.001) were related factors affecting survival. Multivariate regression analysis revealed that age (P=0.001), T stage (P=0.054), N stage (P<0.001), radiotherapy (P=0.010), chemotherapy (P<0.001), bone metastasis (P=0.007), and liver metastasis (P<0.001) were independent factors affecting survival. Moreover, survival analysis was performed on the PSM-matched groups, leading to the following findings: (1) the radiotherapy group exhibited a superior survival prognosis compared with the non-radiotherapy group (P<0.001); (2) the survival prognosis of individuals who underwent radiotherapy and chemotherapy was higher than that of those who underwent chemotherapy alone (P<0.001).

**Conclusion:**

The findings of this study suggest that radiotherapy improves survival benefits for patients with small cell carcinoma of the bladder who undergo bladder-sparing surgery. Furthermore, radiotherapy combined with chemotherapy demonstrates a greater survival benefit compared with chemotherapy alone. The results underscore the importance of considering radiotherapy as a valuable treatment option for such patients, highlighting its potential benefits in improving their overall prognosis.

## Introduction

Bladder cancer encompasses various histological types, with urothelial carcinoma, squamous cell carcinoma, and adenocarcinoma being the most prevalent. Additionally, the relatively rare types include neuroendocrine tumours, carcinosarcoma, and metastatic carcinoma. Among them, urothelial carcinoma is the most frequently observed, accounting for over 90% of bladder cancer cases, followed by squamous cell carcinoma constituting approximately 5%. Neuroendocrine carcinoma is relatively rare, accounting for approximately 1% of the cases, and within this category, small cell neuroendocrine carcinoma is the predominant subtype (1–5). Small cell carcinomas of the bladder typically manifest as large tumours, primarily located in the lateral walls and bottom of the bladder, mainly through lymphatic metastasis. Compared with urothelial carcinoma, small cell carcinoma demonstrates heightened aggressiveness, and early and rapid metastasis, with lymph nodes, liver, bone, lungs, and brain being the most commonly affected sites (6, 7).

The therapeutic approach for small cell carcinoma of the bladder primarily relies on extrapolating treatment strategies from small cell lung cancer and leveraging the knowledge gained from prior experiences in managing this bladder cancer subtype. However, the absence of robust prospective studies and the lack of a standardised treatment strategy contribute to the current uncertainty. Small cell carcinoma of the bladder is extremely aggressive, often presenting with muscular infiltration upon diagnosis, and a significant proportion of patients exhibit distant metastasis (8, 9).

Common treatment approaches for small cell carcinoma of the bladder encompass surgery or radiotherapy combined with neoadjuvant chemotherapy or adjuvant chemotherapy. Surgical options include radical cystectomy, as well as bladder-sparing procedures such as transurethral resection of bladder tumours, transurethral green laser vaporisation of bladder tumours, and partial cystectomy. The chemotherapy regimen for small cell carcinoma of the bladder mirrors that of small cell lung cancer, primarily comprising platinum-based agents. Existing evidence indicates that radical cystectomy plus chemotherapy and chemoradiation therapy are associated with better overall survival compared to monotherapy (10). A study showed that chemotherapy can improve the survival rate of these patients (11). Another research showed that chemotherapy can improve overall survival and radiotherapy is a potential treatment of patients (12). However, a considerable number of patients opt for bladder-preserving surgery even though some of them have distant metastases. Their desire to retain their bladder is strong for various reasons, such as refusing to urinary tract construction, declining quality of life, poor appearance and so on. Currently, there is limited research exploring whether radiotherapy and chemotherapy combined with bladder-preserving surgery could improve survival benefits in these patients. A retrospective study was conducted using the Surveillance, Epidemiology, and End Results (SEER) database to investigate whether bladder-preserving surgery combined with radiotherapy and bladder-preserving surgery combined with radiotherapy and chemotherapy could improve the survival benefits in patients with small cell carcinoma of the bladder.

## Methods

### Study design and study population

**Figure 1:**
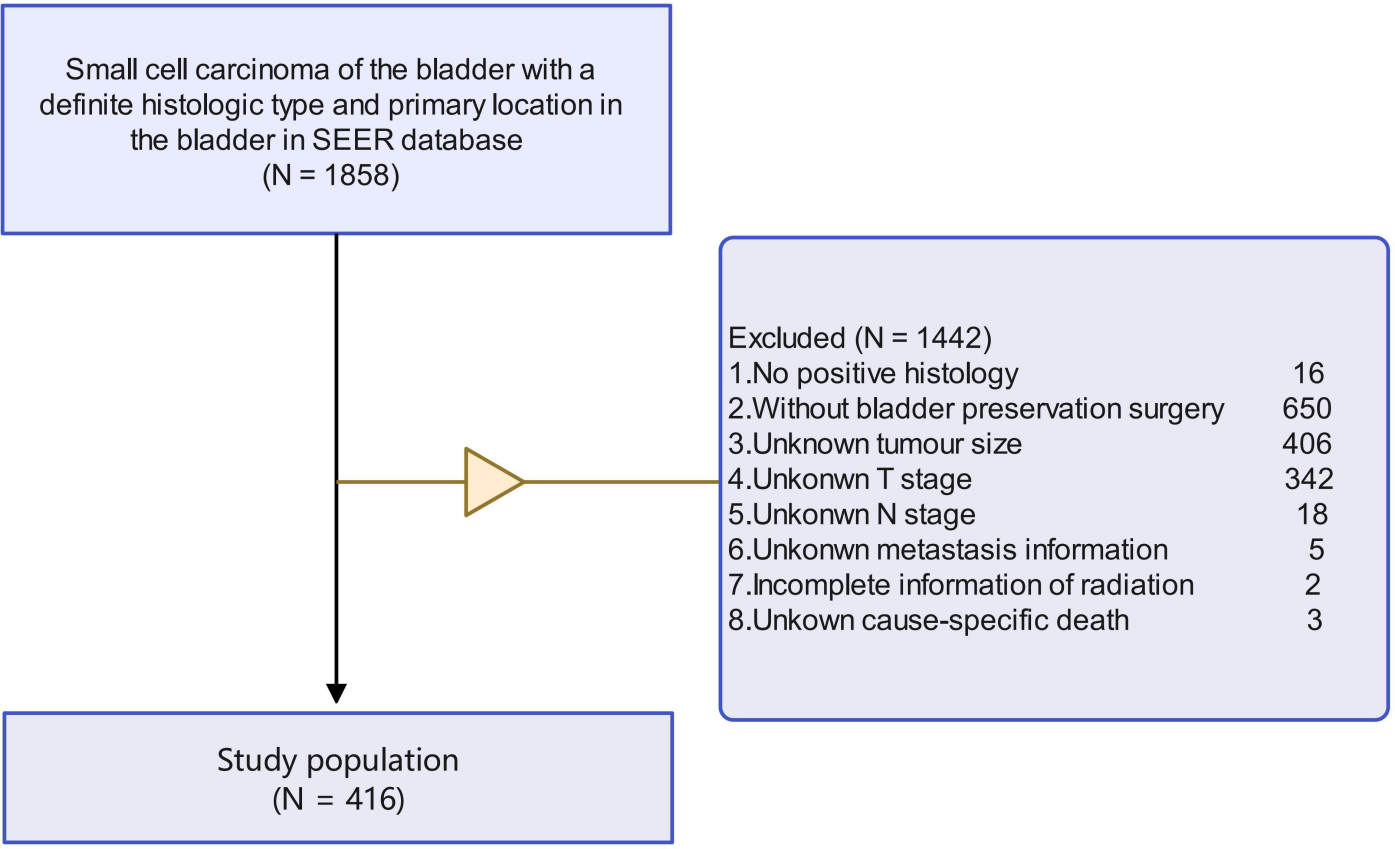
The population’s selection flowchart. **Figure 1**, Population selection Of 1858 patients with small cell carcinoma of the bladder identified in SEER database, and 1442 were excluded for reasons shown.

A comprehensive collection of small cell carcinoma of the bladder data was obtained from the population‐based SEER program of the United States National Cancer Institute. A total of 1858 patients with bladder small cell carcinoma, characterised by specific primary tumour location and histological type and underwent bladder-sparing surgery (transurethral resection of bladder tumours and partial cystectomy), were selected from the SEER database. The time interval of collecting the patient’s data in the SEER database is from 2010 to 2015. The exclusion criteria of this study were as follows: (1) cases that lacked confirmed pathological evidence; (2) those who did not undergo bladder preservation surgery; (3) cases that lacked a definitive American Joint Committee on Cancer (AJCC) stage (seventh edition); incomplete information regarding radiation therapy; exhibited unclear lung, bone, brain, and liver metastases; cases with unknown tumour size and grade; cases with unknown survival outcomes. The population’s selection flowchart is presented in **Figure 1**.

The primary outcome of this study was cancer-specific survival (CSS). The survival time was calculated from the date of diagnosis to the date of death, the last known date when the patient was alive, or the final follow‐up, whichever occurred first. Various covariates were considered, including age (recoded as single ages and 100+), sex, race, tumour stage (T stage) (1-4), node stage (N stage) (0-1), brain metastases, bone metastases, lung metastases, liver metastases, tumour size, radiotherapy information, and chemotherapy information (The choice of chemotherapy drugs are platinum drugs).

### Statistical analysis

Statistical analysis was performed using SPSS software IBM SPSS Statistics version 25.0) and R version 4.1.3 software packages. Statistical significance was set at P<0.05. The numerical variables included in the study were tested for normality using the Shapiro–Wilk method. The results of data conforming to normal distribution are presented as the mean ± standard deviation. The results of non-normally distributed data are presented as quartiles. The distribution of continuous variables was compared by the Wilcoxon rank-sum test or t-test according to the results of the normality test, while the chi-square test was used to compare categorical variables.

The univariate Cox proportional hazard regression analysis was used to identify the high-risk factors associated with CSS. Variables that demonstrated a significance level of P<0.05 were included in the multivariate Cox proportional risk regression analysis. The results are presented as hazard ratio (HR), 95% confidence interval (CI), and P-values.

The R language software was used to conduct a baseline analysis of the clinical parameters of the included patients. Patients were divided into the radiotherapy group or the non-radiotherapy group based on whether they received radiotherapy. The Wilcoxon rank-sum test and chi-square test were used to compare the distribution of clinicopathological features between the two groups.

Additionally, PSM was employed to reduce the potential selection bias resulting from differences in baseline characteristics between the two groups. Variables with a significance level of P<0.05 were matched using PSM, and the propensity score for each patient was calculated. A 1:1 neighbor ratio matching was conducted between the two groups, with a caliper matching threshold of 0.02 (Nearest neighbor matching within caliper). Subsequently, Kaplan–Meier survival analysis was performed to assess the effects of radiotherapy on CSS before and after PSM. The same method was applied to evaluate the effect of radiotherapy combined with chemotherapy on CSS.

## Results

### Population characteristics

The patient characteristics are presented in **Table 1**. Among the 416 patients with small cell carcinoma of the bladder, the median age was 76 (interquartile range: 67-84) years. Of the patients, 76.9% were males, and 23.1% were females. Most patients were Caucasian (91.3%), with 6.2% being black and 2.4% belonging to other racial groups. Regarding the T stage, 7% had T0 (TIS), 20.4% had T1, 63.9% had T2, 6.0% had T3, and 8.9% had T4). In terms of the N stage, 80.3% had N0 disease and 19.7% had N1 disease. Among the patients, 10.1% had bone metastases, while 89.9% did not. Only 1% had brain metastases, while 99% did not. Liver metastases were observed in 14.7% of the patients, while 85.3% did not have liver metastases. Lung metastases were observed in 4.3% of the patients, while 95.7% did not have liver metastases. The median tumour size was 7.0 (interquartile range: 4-11) cm, and the proportion of tumour-specific deaths was 65.9%. The median survival time for the patients was 10.5 (interquartile range: 4-32.25) months. Among the patients, 36.1% received postoperative radiotherapy, while 63.9% did not. Furthermore, 61.3% received chemotherapy, while 38.7% did not. All patients underwent bladder-preserving surgery.

**Table 1:** Population characteristics.

Characteristics	Total (n=416)
Age	
Years(median[IQR])	76.00[67.00,84.00]
Race(%)	
Black	26(6.2)
White	380 (91.3)
Others	10 (2.4)
Sex(%)	
Male	320 (76.9)
Female	96 (23.1)
T stage(%)	
Tis	3 (0.7)
T1	85(20.4)
T2	266 (63.9)
T3	25 (6.0)
T4	37 (8.9)
N stage(%)	
N0	334 (80.3)
N1	82(19.7)
Bone metastases(%)	
Yes	42 (10.1)
No	374 (89.9)
Liver metastases(%)	
Yes	61 (14.7)
No	355 (85.3)
Lung metastases(%)	
Yes	18(4.3)
No	398 (95.7)
Brain metastases(%)	
Yes	4(1.0)
No	412 (99.0)
Tumor size	
Centimetre(median[IQR])	7.00[4.00,11.00]
Time	
Months(median[IQR])	10.50[4.00,32.25]
Radiotherapy	
Yes	150(36.1)
No	266 (63.9)
Chemotherapy	
Yes	255(61.3)
No	161 (38.7)

IQR, interquartile range; T stage, tumour stage; N stage, node stage.

### Related factors affecting survival

Based on univariate regression analysis, several factors were found to significantly affect survival, including age (HR, 95% CI, P), T stage, N stage, chemotherapy, bone metastasis, liver metastasis, lung metastasis, tumour size, and radiotherapy. However, variables such as sex, race, and brain metastasis were not associated with CSS. Subsequently, a multivariate analysis was performed, considering, age, T stage, N stage, chemotherapy, bone metastasis, liver metastasis, lung metastasis, tumour size, and radiotherapy. It was found that age, T stage, N stage, radiotherapy, chemotherapy, bone metastasis, and liver metastasis were independent factors affecting survival (**Table 2**).

**Table 2:** Factors associated with CSS in patients with small cell bladder cancer.

	Univariate analysis			Multivariate analysis		
Covariate	HR	95%CI	*p*	HR	95%CI	*p*
Age (years)						
	1.02	1.01-1.03	0.000*	1.02	1.01-1.03	0.001*
Sex						
	1.07	0.80-1.41	0.660			
Race						
	0.93	0.62-1.40	0.730			
T stage						
	1.23	1.06-1.42	0.005*	1.16	1.00-1.34	0.054*
N stage						
	2.11	1.6-2.78	0.000*	2.05	1.50-2.80	0.000*
Bone metastases						
	2.65	1.85-3.82	0.000*	1.74	1.16-2.59	0.007*
Liver metastases						
	3.17	2.34-4.32	0.000*	2.76	1.89-4.03	0.000*
Lung metastases						
	2.20	1.27-3.78	0.005*	1.03	0.56-1.90	0.928
Brain metastases						
	2.11	0.78-5.68	0.140			
Tumor size						
	1.05	1.01-1.08	0.005*	1.02	0.98-1.05	0.313
Radiotherapy						
	0.52	0.40-0.67	0.000*	0.70	0.53-0.92	0.010*
Chemotherapy						
	0.52	0.41-0.66	0.000*	0.48	0.36-0.64	0.000*

CSS, cancer-specific survival; HR, hazard ratio, 95%CI, 95% confidence interval; T stage, tumour stage; N stage, node stage; p, P value.

The meaning of the symbol * is having statistical significance.

In our sample group, out of 416 patients, 150 patients received postoperative radiotherapy while 266 did not. A comparison of differences between these two groups revealed that chemotherapy and tumour size (P-value) exhibited significant statistical significance. Subsequently, based on these factors, 125 matching pairs (250 patients) were included in the PSM post queue. The matched data demonstrated that there were no statistical differences between the two groups in terms of tumour size and radiotherapy. Moreover, various variables also exhibited good consistency between the matched groups (**Table 3**).

**Table 3:** Characteristics of radiotherapy and non-radiotherapy groups in the before and after PSM cohorts.

	Before PSM cohort (n=416)				After PSM cohort (n=250)				
Characteristics	T(n=416)	NRT(n=266)	RT(n=150)	*P*	T(n=250)	NRT(n=125)	RT(n=125)	*p*	SMD
**Age**				0.102				0.640	0.021
Years (median[IQR])	416	77.0[68.0,84.0]	74.5[66.0,83.0]		250	75.0[66.0,82.0]	75.0[65.0,83.0]		
**Race** (%)				0.645				0.415	0.168
Black	26	17(6.4)	9 (6.0)		19	11(8.8)	8 (6.4)		
White	380	244(91.7)	136 (90.7)		224	112(89.6)	112(89.6)		
Others	10	5(1.9)	5 (3.3)		7	2(1.6)	5 (4.0)		
**Sex** (%)				0.450				1.000	0.020
Male	320	201(75.6)	119 (79.3)		201	101(80.8)	100 (80.0)		
Female	96	65(24.4)	31 (20.7)		49	24(19.2)	25 (20.0)		
**T stage (%)**				0.680				0.592	0.213
Tis	3	1 (0.4)	2 (1.3)		1	0 (0.0)	1 (0.8)		
T1	85	54 (20.3)	31 (20.7)		47	21(16.8)	26 (20.8)		
T2	266	174 (65.4)	92 (61.3)		160	84 (67.2)	76 (60.8)		
T3	25	16(6.0)	9 (6.0)		16	9(7.2)	7(5.6)		
T4	37	21 (7.9)	16 (10.7)		26	11 (8.8)	15(12.0)		
**N stage (%)**				0.313				1.000	0.019
N0	334	218(82.0)	116(77.3)		193	96(76.8)	97(77.6)		
N1	82	48(18.0)	34(22.7)		57	29(23.2)	28(22.4)		
**Bone metastases(%)**				1.000				0.701	0.073
Yes	42	27(10.2)	15(10.0)		31	17(13.6)	14(11.2)		
No	374	239(89.8)	135(90.0)		219	108(86.4)	111(88.8)		
**Liver metastases(%)**				0.061				0.075	0.250
Yes	61	46(17.3)	15(10.0)		37	24(19.2)	13(10.4)		
No	355	220(82.7)	135(90.0)		213	101(80.8)	112(89.6)		
**Lung metastases(%)**				0.133				0.497	0.129
Yes	18	15(5.6)	3(2.0)		9	6(4.8)	3(2.4)		
No	398	251(94.4)	147(98.0)		241	119(95.2)	122(97.6)		
**Brain metastases(%)**				0.268				0.245	0.222
Yes	4	1(0.4)	3(2.0)		3	0(0.0)	3(2.4)		
No	412	265(99.6)	147(98.0)		247	125(100.0)	122(97.6)		
**Tumor size**				<0.001*				1.000	<0.001
Centimetre (median[IQR])	416	11.0[5.0,11.0]	5.0[3.0,11.0]		250	6.0[4.0,11.0]	6.0[4.0,11.0]		
**Chemotherapy**				<0.001*				1.000	<0.001
Yes	255	134(50.4)	121(80.7)		196	98(78.4)	98(78.4)		
No	161	132(49.6)	29(19.3)		54	27(21.6)	27(21.6)		

PSM, propensity score matching; RT, radiotherapy; NRT, without radiotherapy; IQR, interquartile range; T stage, tumour stage; N stage, node stage; p, P value; SMD, STD Mean Difference.

### Effects of radiotherapy vs. non-radiotherapy on CSS

**Figure 2:**
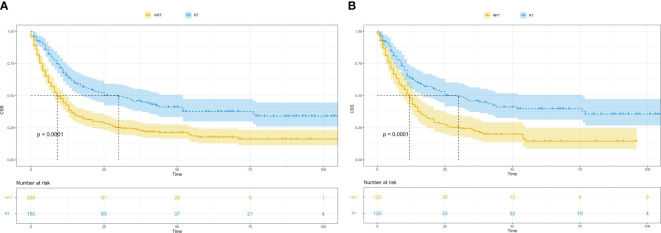
Kaplan-Meier survival curves in patients with and without radiotherapy. **(A)**, Kaplan-Meier survival curves in patients with and without radiotherapy before PSM; CSS, cancer-specific survival; RT, radiotherapy; NRT, without radiotherapy; PSM, propensity score matching. **(B)**, Kaplan-Meier survival curves in patients with and without radiotherapy after PSM; CSS, cancer-specific survival; RT, radiotherapy; NRT, without radiotherapy; PSM, propensity score matching.

Survival analysis was conducted to compare tumour-specific survival between the radiotherapy group and the non-radiotherapy group. The findings revealed a significantly higher outcome and prognosis in the radiotherapy group compared with the non-radiotherapy group (Before PSM, P<0.0001; After PSM, P<0.0001) (**Figure 2**).

Additionally, a PSM analysis was performed to evaluate the effects of radiotherapy combined with chemotherapy vs. chemotherapy alone.Within our sample group, 121 patients received postoperative chemotherapy combined with radiotherapy, while 134 patients received chemotherapy alone. Comparisons between these two groups revealed significant statistical significance in terms of liver metastasis and tumour size (P-value). Consequently, based on these factors, 90 matching pairs (180 patients) were included in the PSM post queue. The matched data demonstrated no statistically significant differences in terms of liver metastasis and tumour size between the two groups. Moreover, various variables exhibited good consistency between the matched groups (**Table 4**).

**Table 4:** Characteristics of radiotherapy combined with chemotherapy and chemotherapy alone groups in the before and after PSM cohorts.

	Before PSM cohort (n=255)				After PSM cohort (n=180)				
Characteristics	Total(n=255)	CT without RT(n=134)	RT with CT(n=121)	*p*	Total(n=180)	CT without RT(n=90)	RT with CT(n=90)	*p*	SMD
**Age**				0.932				0.696	0.093
Years (median[IQR])	255	73.0[66.0,79.0]	73.0[64.0,82.0]		180	75.0[66.0,79.0]	73.0[64.0,81.8]		
**Race** (%)				0.269				0.529	0.169
Black	17	11(8.2)	6 (5.0)		15	9(10.0)	6(6.7)		
White	231	121(90.3)	110 (90.9)		159	79(87.8)	8088.9)		
Others	7	2(1.5)	5 (4.1)		6	2(2.2)	4 (4.4)		
**Sex** (%)				0.428				0.852	0.056
Male	200	102(76.1)	98 (81.0)		144	71(78.9)	73 (81.1)		
Female	55	32(23.9)	23 (19.0)		36	19(21.1)	17 (18.9)		
**T stage (%)**				0.624				0.537	0.221
Tis	1	0 (0.0)	1(0.8)		0	0 (0.0)	0 (0.0)		
T1	52	25 (18.7)	27 (22.3)		37	15(16.7)	22 (24.4)		
T2	159	83 (61.9)	76(62.8)		112	57 (63.3)	55(61.1)		
T3	20	12(9.0)	8 (6.6)		14	8(8.9)	6(6.7)		
T4	23	14 (10.4)	9 (7.4)		17	10 (11.1)	7(7.8)		
**N stage(%)**				0.443				0.469	0.135
N0	198	101(75.4)	97(80.2)		151	68(75.6)	73(81.1)		
N1	57	33(24.6)	24(19.8)		39	22(24.4)	17(18.9)		
**Bone metastases(%)**				0.372				1.000	0.035
Yes	29	18(13.4)	11(9.1)		21	11(12.2)	10(11.1)		
No	226	116(86.6)	110(90.9)		159	79(87.8)	80(88.9)		
**Liver metastases(%)**				0.003*				1.000	<0.001
Yes	40	30(22.4)	10(8.3)		16	8(8.9)	8(8.9)		
No	215	104(77.6)	111(91.7)		164	82(91.1)	82(91.1)		
**Lung metastases(%)**				0.128				0.613	0.151
Yes	13	10(7.5)	3(2.5)		4	1(1.1)	3(3.3)		
No	242	124(92.5)	118(97.5)		176	89(98.9)	87(96.7)		
**Brain metastases (%)**				0.544				0.244	0.263
Yes	4	1(0.7)	3(2.5)		3	0(0.0)	3(3.3)		
No	251	133(99.3)	118(97.5)		177	90(100.0)	87(96.7)		
**Tumor size**				0.009*				1.000	<0.001
Centimetre (median[IQR])	255	8.0[4.0,11.0]	5.0[3.0,11.0]		180	7.0[4.0,11.0]	7.0[4.0,11.0]		

PSM, propensity score matching; IQR, interquartile range; RT with CT, radiotherapy combined with chemotherapy; CT without RT, chemotherapy without radiotherapy; T stage, tumour stage; N stage, node stage; p, P value; SMD, STD Mean Difference.

### Effects of radiotherapy combined with chemotherapy vs. chemotherapy alone on CSS

**Figure 3:**
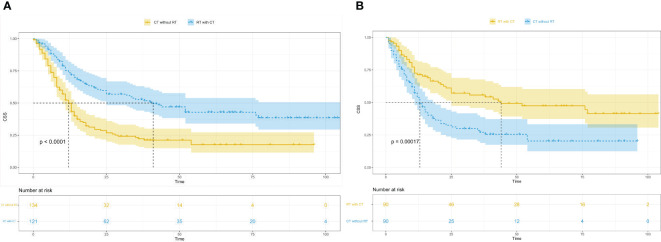
Kaplan-Meier survival curves in patients of radiotherapy combined with chemotherapy and the patients of chemotherapy alone. **(A)**, Kaplan-Meier survival curves in patients of radiotherapy combined with chemotherapy and the patients of chemotherapy alone before PSM; CSS, cancer-specific survival; RT with CT, radiotherapy combined with chemotherapy; CT without RT, chemotherapy without radiotherapy; PSM, propensity score matching. **(B)**, Kaplan-Meier survival curves in patients of radiotherapy combined with chemotherapy and the patients of chemotherapy alone after PSM; CSS, cancer-specific survival; RT with CT, radiotherapy combined with chemotherapy; CT without RT, chemotherapy without radiotherapy; PSM, propensity score matching.

Survival analysis was conducted to compare tumour-specific survival between the radiotherapy combined with chemotherapy group and the chemotherapy alone group. The results revealed a significantly higher outcome and prognosis in the radiotherapy combined with chemotherapy group compared with the chemotherapy alone group (Before PSM, P<0.0001; After PSM, P=0.00017) (**Figure 3**).

## Discussion

Small cell carcinoma of the bladder is a rare and fatal disease. Currently, cystectomy remains the standard local treatment due to its high metastatic potential. However, in clinical practice, many patients require bladder preservation for various reasons. The treatment strategy and therapeutic effect for this specific patient population remain poorly understood. A previous approach for bladder preservation involves the use of platinum-etoposide chemotherapy combined with bladder radiotherapy. However, due to the relatively small sample size, the therapeutic effect of this approach remains unclear, necessitating further investigation (13).

In this study, the effect of radiotherapy and chemotherapy on tumour-specific survival in patients with small cell carcinoma with bladder preservation was investigated using a larger sample size. The PSM algorithm was used to balance the groups and address potential confounding factors, thereby improving the reliability of our findings. Univariate Cox regression analysis revealed that age, T stage, N stage, chemotherapy, bone metastasis, liver metastasis, lung metastasis, tumour size, and radiotherapy were factors affecting patient survival. Subsequent multivariate Cox regression analysis revealed that age, T stage, N stage, radiotherapy, chemotherapy, bone metastasis, and liver metastasis were independent predictors of CSS. Kaplan–Meier survival analysis demonstrated that postoperative radiotherapy improved survival outcomes in patients with bladder-sparing small cell carcinoma compared with the non-radiotherapy group. Furthermore, when comparing the group receiving chemotherapy combined with radiotherapy to the group receiving chemotherapy alone, the former exhibited a significant improvement in CSS. Based on our study findings, it can be concluded that postoperative radiotherapy and chemotherapy combined with radiotherapy may be potential protective factors for CSS in patients with small cell carcinoma of the bladder.

Small cell carcinoma of the bladder is an extremely rare malignancy, accounting for <1% of all bladder tumours. Its prognosis is very poor due to its highly aggressive behaviour and highly metastatic potential (14). Our study corroborated previous findings that bone and liver metastases were more prevalent in patients with distant metastases of small cell carcinoma of the bladder, whereas brain and lung metastases were relatively rare (15). The findings of our study revealed that age, T stage, N stage, chemotherapy, bone metastasis, liver metastasis, lung metastasis, tumour size, and radiotherapy were factors affecting patient survival, among which age, T stage, N stage, radiotherapy, chemotherapy, bone metastasis, and liver metastasis were independent risk factors affecting CSS. These findings emphasise the importance of not only improving early disease screening but also prioritising systemic treatment in patients with small cell carcinoma of the bladder, as systemic interventions carry greater significance in disease control compared with local treatment approaches.

Lohrisch et al. conducted a retrospective analysis of 14 patients with small cell carcinoma of the bladder. Their study demonstrated that integrated chemoradiation for patients with limited stage SCCB generates a realistic expectation of long term survival, but prospective trials are needed to confirm this view (16). Similarly, Richard et al. retrospectively analysed 27 patients with limited disease (Tx-4N0-1M0) small cell carcinoma of the bladder who received chemotherapy combined with radiotherapy. Their findings indicated that this combined treatment approach yielded favourable efficacy and a high bladder retention rate (17). In another retrospective analysis by Caroline et al., patients with small cell carcinoma of the bladder from 26 institutions in the United Kingdom were examined. The study revealed an overall poor prognosis for small cell carcinoma of the bladder; however, it also demonstrated that chemotherapy could improve the survival benefits for patients (15). Hiroko et al. conducted a retrospective analysis involving 12 Japanese patients with small cell carcinoma of the bladder, suggesting that radiotherapy is a potential treatment option. The study further indicated that systemic chemotherapy combined with local radiotherapy might effectively improve survival outcomes (12). Consistent with these prior studies, our findings support the notion that radiotherapy and chemotherapy combined with radiotherapy could improve the survival prognosis and CSS in patients with small cell carcinoma of the bladder.

Previous studies have reported that radiotherapy and chemotherapy combined with radical cystectomy could improve the patient survival rate (10, 12, 18). Curtis et al. analysed the case data of 11 patients with small cell carcinoma of the bladder, all of whom underwent transurethral resection of the bladder tumour, followed by induction chemotherapy, consolidation radiotherapy, or concurrent chemoradiotherapy. The results revealed that radiotherapy and chemotherapy could effectively prevent recurrence and protect bladder function, even in patients with locally advanced disease (19). Similarly, the study conducted by Christian et al. suggested that radiotherapy or chemoradiotherapy with selective bladder preservation following transurethral resection of bladder tumours is a viable alternative for patients with high-risk bladder cancer, serving as an alternative to intravesical treatment or early cystectomy (20). Our findings are consistent with those of previous studies. In this retrospective analysis involving 416 patients with small cell carcinoma of the bladder who underwent bladder-sparing surgery, it was observed that postoperative radiotherapy and chemotherapy combined with radiotherapy could improve the survival benefits of patients with small cell carcinoma of the bladder.

### Advantages and limitations of this paper

With a relatively large sample size and the use of statistical methods such as PSM, this study effectively eliminated bias and confounding factors, thus maximising the authenticity of the findings. The conclusion of this paper offers a potential alternative treatment strategy for patients who refuse to undergo radical cystectomy. Radiotherapy and chemotherapy after bladder-sparing surgery might improve the survival benefits of patients, consistent with previous studies. However, it is important to note that this study and previous studies were retrospective in nature. Our research conclusion comes from the database and has limitations, emphasising the need for future prospective, multicentre, randomised studies to evaluate the effect of surgery or radiotherapy after neoadjuvant chemotherapy. And we hope that our study can provide a research direction, provide some reference value for it. By analysing the factors influencing the survival benefits of patients, it was concluded that early screening and systemic treatment of this disease should be prioritised rather than relying solely on local surgical resection. Additionally, given the poor prognosis associated with this tumour type, further research is warranted to determine optimal treatment plans and explore novel molecular markers. Such investigations hold the potential for achieving early diagnosis and improved prognosis.

## Conclusion

In conclusion, for patients with small cell carcinoma of the bladder undergoing bladder-sparing surgery, radiotherapy alone and radiotherapy combined with chemotherapy could improve the survival benefits, and radiotherapy might offer greater benefits for these patients.

## Data availability statement

The datasets presented in this study can be found in online repositories. The names of the repository/repositories and accession number(s) can be found below: https://seer.cancer.gov/.

## Ethics statement

Ethical approval was not required for the study involving humans in accordance with the local legislation and institutional requirements. Written informed consent to participate in this study was not required from the participants or the participants’ legal guardians/next of kin in accordance with the national legislation and the institutional requirements.

## Author contributions

FL: Conceptualization, Formal Analysis, Methodology, Writing – original draft, Writing – review & editing. FZ: Formal Analysis, Software, Writing – original draft. XQ: Formal Analysis, Software, Writing – original draft. LQ: Formal Analysis, Software, Writing – original draft. JW: Data curation, Writing – original draft. CD: Data curation, Writing – original draft. YK: Visualization, Writing – review & editing. XZ: Conceptualization, Writing – review & editing. LF: Conceptualization, Writing – review & editing.
